# Language of Perseverative Thoughts Predicts Emotion Regulation Strategy Choice

**DOI:** 10.1007/s42761-026-00357-w

**Published:** 2026-03-07

**Authors:** Yiqin Zhu, Kevin Trent, Beatris Garcia, Renee J. Thompson

**Affiliations:** Department of Psychological and Brain Sciences, Washington University in St. Louis, St Louis, MO USA

**Keywords:** Perseverative thought, Emotion regulation choice, LIWC, Natural language processing, Experience sampling

## Abstract

A growing body of research has sought to understand the contextual factors that influence emotion regulation (ER) strategy choice. As a process that is usually involuntary to start and difficult to end, perseverative thought (PT) can be an ongoing context that influences how individuals respond to emotions. This study investigated how different PT dimensions, captured through written language, predicted subsequent ER strategy choice, including engagement (i.e., reappraisal, acceptance, problem-solving) and disengagement (i.e., experiential avoidance, distraction, expressive suppression) strategies. College students (*N* = 413) completed an experimental paradigm once a day for ten days. Each night, they underwent a PT induction writing task and were instructed to regulate (to feel better) and report their ER strategy use (1 = selected; 0 = not selected). We used the Linguistic Inquiry Word Count program to construct PT dimensions and multilevel logistic regressions to examine links between each PT dimension and subsequent ER strategy selection. Within individuals, higher negative valence during PT predicted a higher likelihood of selecting distraction. Higher self-focus during PT predicted a greater likelihood of selecting experiential avoidance and expressive suppression. Higher situation certainty, more social words, and more past focus during PT predicted a lower likelihood of selecting problem solving. Overall, the findings support the role of PT dimensions as important contextual features for ER strategy selection and highlight the value of computational linguistics and a dimensional approach for studying PT.

**Supplementary Information:**

The online version contains supplementary material available at 10.1007/s42761-026-00357-w.

The selection or choice of emotion regulation (ER) strategies has been identified as an important stage of ER (Gross, 2015) and has been linked to psychopathology like major depressive disorder (MDD; e.g., Liu & Thompson, 2017). Thus, understanding precipitants of ER choice is critical, especially for understanding how psychopathology develops. Growing lines of research have sought to understand the contextual factors that influence ER selection, including cognitive (Sheppes et al., 2014), emotional (Sheppes et al., 2011), and social (English et al., 2017) contexts. 

One untested cognitive context is perseverative thought (PT), a thought process that is repetitive, uncontrollable, and focused on negative content (Ehring & Watkins, 2008). As a robust risk factor for internalizing disorders and emotion dysregulation (e.g., Ruscio et al., 2011), PT can be counterproductive, as it repeatedly produces and sustains negative affect (Kircanski et al., 2018; Moberly & Watkins, 2008), to which individuals need to respond. More importantly, PT often starts automatically and involuntarily (Watkins & Roberts, 2020), and individuals, especially those at risk for internalizing psychopathology, usually have difficulty disengaging from PT (as reflected in its definition of uncontrollability; Koster et al., 2011). Thus, PT can be an ongoing context when individuals make different ER choices. Recent work has demonstrated the promise of psycholinguistics tools to assess PT that is covert, multidimensional, and dynamic (Stade et al., 2023). The current study aimed to examine the ways that PT dimensions, captured through written language, predicted subsequent ER strategy choice.

In clinical psychological science, PT has been conceptualized within diagnostic and trans-diagnostic frameworks. For example, rumination has been primarily studied in MDD and worry in anxiety disorders (Hong, 2007). However, others have operationalized PT independent of diagnostic contexts, arguing that these separate categorical constructs share common characteristics (e.g., repetitiveness), which explain the majority of the variance in their associations with psychopathology (Ehring & Watkins, 2008; Spinhoven et al., 2015).

In addition to repetitiveness, studies have identified other potentially salient dimensions underlying PT (Segerstrom et al., 2016; Szkodny & Newman, 2019). We and others have argued that such a transdiagnostic and dimensional approach is critical to isolate targetable dimensions underlying the association between PT and ER deficits (e.g., Hankin et al., 2016; McLaughlin & Sheridan, 2016). In choosing which PT dimensions to include, we followed Hallion et al. (2022), who empirically derived a five-factor model of PT, including dimensions of valence, self-focus, situational certainty, interpersonal, and dyscontrol. In addition to these, we examined discrepancy and temporal orientation as potentially salient facets of PT that could be indexed by written language. We described definitions of each PT dimension in Table 1.

**Table 1:** Dimensions of perseverative thought (PT) and corresponding linguistic features

PT Dimensions	Definition	Tool	Linguistic Features	Example Words
Negative Valence	The degree of negative sentiment of the PT thought content	LIWC	Negative tone	bad, wrong, too much, hate
Self-Focus	An awareness of self-referent, internally generated information that stands in contrast to an awareness of externally generated information derived through sensory receptors. (Ingram, 1990, p.156)	LIWC	First-person singular pronouns	I, me, my, myself
Discrepancy	Mental comparisons between actual states and ideal states (including behaviors, emotions, goals, and events)	LIWC	Discrepancy	would, can, want, could
Situational Certainty	Impressions of certainty of external situations/events	LIWC	Certitude	really, actually, of course, real
Interpersonal	Thought content related to interpersonal processes (e.g., interpersonal stressors, conflicts, etc.) during PT	LIWC	Social	fight, tell, he, she
Temporal Orientation	The extent to which thoughts are past, present, or non-present-focused	LIWC	Past-focus	was, had, were, been
		LIWC	Future-focus	will, going to, have to, may
Dyscontrol	The extent to which individuals were unable to disengage from negative thoughts during PT	Cosine between Sentence Embeddings	Repetitiveness	not applicable

*LIWC *Linguistic Inquiry and Word Count program (Boyd et al., 2022)

Although PT has been increasingly conceptualized as an ER choice itself, it has been argued that, due to PT’s uncontrollable nature (e.g., Vine, 2023), it is better conceptualized as a process of attentional control failure (Singh et al., 2025; Whitmer & Gotlib, 2013; Zetsche et al., 2018). The current investigation, exploring whether PT can be a potential cognitive context of ER choice, followed the latter conceptualization. Evidence hinting at the associations between PT and subsequent ER strategy choices was from findings on between-individual correlations among ER strategies. In a meta-analysis, Naragon-Grainey et al. (2017) found that individuals who reported more (versus less) habitual rumination and worry reported greater use of disengagement strategies (e.g., distraction) as opposed to engagement strategies (e.g., reappraisal). This trait-level evidence supports that PT might relate to regulatory attempts to avoid negative thoughts or experiences. However, such between-individual findings do not always generalize to the within-individual level (Fisher et al., 2018). For example, the within-person factor structure of ER strategies is different from the between-person structure (McMahon & Naragon-Gainey, 2019). Therefore, research is needed to address how individuals engage in different regulatory behaviors *when* PT creates and sustains negative emotional states.

Given PT’s multi-dimensional nature, it is also important to examine how such associations differ across specific dimensions of PT. For example, the temporal orientation of PT (i.e., past vs. future focused) may differentially influence ER choice regardless of how negative or repetitive PT is. A more past-focused PT could be associated with a decreased likelihood of selecting problem-solving, as past events are viewed as unchangeable. In contrast, a more future-focused PT might show the opposite within-individual association. This dimensional approach allows us to examine specific, isolated facets of PT, and parse their associations with subsequent ER choice. This could serve to identify particularly salient dimensions of PT that could be targeted by interventions.

Linguistic tools, through their ability to reveal cognitive and emotional patterns in a non-intrusive way, are valuable in assessing dynamic processes (Stade et al., 2023), especially those that are challenging to assess via self-report due to their complex nature and demand for self-awareness. As presented in Table 1, several linguistic features (e.g., certitude) can be used as proxies for PT dimensions (e.g., situational certainty). Research has linked these dimensions to certain psychological constructs in other contexts, such as affective state (Corbin et al., 2023), temporal focus (Pennebaker & Stone, 2003), and self-focused attention in depression and anxiety (Brockmeyer et al., 2015). In the present study, participants provided their thought content during an experimental PT-induction paradigm that was administered for ten consecutive nights. Therefore, these linguistic markers were PT-specific and used as proxies for PT dimensions, including those difficult to assess with self-report. For example, the repetitiveness dimension, which usually requires meta-cognitive appraisal in self-report, can be captured through sentence embeddings in natural language processing (Iter et al., 2018).

We examined how PT dimensions assessed using linguistic features were associated with subsequent ER strategy selection within individuals in a college sample. College students usually show elevated levels of internalizing psychopathology (Basterfield et al., 2024) and high levels of PT (Davey et al., 2022) and are thus a suitable sample for this investigation. We used seven linguistically measured PT dimensions (i.e., valence, self-focus, interpersonal, discrepancies, situational certainty, temporal orientation, repetitiveness) and six ER strategies. Consistent with Naragon-Gainey et al. (2017), we grouped the six ER strategies into two categories of engagement (i.e., reappraisal, acceptance, problem-solving) and disengagement (i.e., experiential avoidance, distraction, expressive suppression). We aimed to test how a specific PT dimension predicted an ER choice (see Table 2 for every specific pre-registered hypothesis; some of them were exploratory; Aim 1). We also aimed to test our hypotheses about PT dimensions predicting ER choice in bigger categories (Aim 2), that higher levels of (a) negative valence, (b) self-focus, (c) discrepancy, and (d) repetitiveness in PT language would predict a greater likelihood of selecting disengagement ER strategies and a decreased likelihood of selecting engagement strategies. Finally, we tested our overall hypothesis that PT dimensions were contextual predictors of ER choice (Aim 3).

**Table 2:** Associations between perseverative thoughts dimensions and selection of engagement emotion regulation strategies at the within-individual level

Disengagement Strategies	Experiential Avoidance					Expressive Suppression					Distraction				
	Hyp	OR	*SE*	95% CI	*p*	Hyp	OR	*SE*	95% CI	*p*	Hyp	OR	*SE*	95% CI	*p*
1. Negative Valence	**+ (>1)**	1.26	0.21	[0.91, 1.74]	.16	**+ (>1)**	1.04	0.09	[0.88, 1.22]	.64	**+ (>1)**	**1.25**	**0.13**	**[1.02, 1.52]**	**.032**
2. Self-Focus	**+ (>1)**	**1.36**	**0.21**	**[1.004, 1.85]**	**.047**	**+ (>1)**	**1.21**	**0.10**	**[1.02, 1.44]**	**.025**	**+ (>1)**	1.16	1.11	[0.96, 1.40]	.13
3. Discrepancies	**+ (>1)**	1.00	0.15	[0.75, 1.31]	1.00	**+ (>1)**	0.91	0.07	[0.78, 1.05]	.20	**+ (>1)**	0.99	0.09	[0.83, 1.19]	.94
4. Certitude	**+ (>1)**	**0.76**	**0.10**	**[0.58, 0.99]**	**.040**	**+ (>1)**	1.01	0.08	[0.87, 1.18]	.88	**+ (>1)**	1.17	0.11	[0.97, 1.40]	.10
5. Interpersonal	**E**	0.96	0.14	[0.71, 1.28]	.77	**E**	**1.23**	**0.11**	**[1.04, 1.45]**	**.018**	**E**	0.94	0.09	[0.78, 1.13]	.50
6. Past Focus	**+ (>1)**	0.97	0.13	[0.74, 1.27]	.83	**+ (>1)**	1.01	0.08	[0.86, 1.17]	.94	**+ (>1)**	1.15	0.10	[0.96, 1.37]	.13
7. Future Focus	**E**	**1.49**	**0.27**	**[1.05, 2.11]**	**.020**	**E**	1.15	0.10	[0.98, 1.36]	.09	**E**	0.93	0.09	[0.77, 1.12]	.44
8. Repetitiveness	**+ (>1)**	1.12	0.20	[0.78, 1.59]	.64	**+ (>1)**	0.99	0.08	[0.85, 1.16]	.93	**+ (>1)**	1.00	0.09	[0.84, 1.19]	1.00
Engagement Strategies	Reappraisal					Problem solving					Acceptance				
	Hyp	OR	SE	95% CI	*p*	Hyp	OR	SE	95% CI	*p*	Hyp	OR	SE	95% CI	*p*
1. Negative Valence	**– (<1)**	0.91	0.11	[0.71, 1.16]	.46	**– (<1)**	1.15	0.09	[0.98, 1.34]	.08	**– (<1)**	0.94	0.07	[0.81, 1.10]	.46
2. Self-Focus	**– (<1)**	0.92	0.12	[0.70, 1.20]	.54	**– (<1)**	1.11	0.09	[0.95, 1.29]	.19	**– (<1)**	0.90	0.07	[0.77, 1.05]	.19
3. Discrepancies	**– (<1)**	1.02	0.13	[0.79, 1.32]	.90	**– (<1)**	1.03	0.08	[0.88, 1.19]	.74	**– (<1)**	1.00	0.08	[0.86, 1.16]	.99
4. Certitude	**+ (>1)**	0.90	0.11	[0.71, 1.14]	.38	**– (<1)**	**0.86**	**0.06**	**[0.75, 0.997]**	**.046**	**+ (>1)**	0.98	0.07	[0.85, 1.13]	.80
5. Interpersonal	**E**	**0.74**	**0.09**	**[0.58, 0.95]**	**.019**	**E**	**0.66**	**0.06**	**[0.56, 0.78]**	**<.001**	**E**	0.97	0.08	[0.83, 1.14]	.73
6. Past Focus	**+ (>1)**	0.90	0.10	[0.72, 1.13]	.38	**– (<1)**	**0.74**	**0.06**	**[0.64, 0.86]**	**.023**	**+ (>1)**	1.10	0.08	[0.94, 1.27]	.23
7. Future Focus	**– (<1)**	1.03	0.15	[0.79, 1.37]	.77	**+ (>1)**	1.11	0.06	[0.94, 1.32]	.22	**– (<1)**	1.01	0.08	[0.87, 1.19]	.86
8. Repetitiveness	**– (<1)**	**1.27**	**0.15**	**[1.00, 1.60]**	**.046**	**– (<1)**	1.15	0.09	[0.99, 1.33]	.07	**– (<1)**	1.01	0.07	[0.88, 1.17]	.84

“+ (> 1)” denotes significant positive associations where OR is larger than 1 in hypotheses, “– (< 1)” denotes significant negative associations where OR is less than 1 in hypotheses, “E” denotes Exploratory in hypotheses

*Hyp *hypothesis, *OR *odds ratio, *SE *standard error of odds ratio. 95% CI = 95% Confidence Interval of Odds Ratio; Coefficients were calculated based on standardized data

## Method

### Transparency and Openness

The study aims, hypotheses, and analytic plan for this secondary data analysis paper were pre-registered (https://osf.io/zu68k/?view_only=93938cf95ded47eeaa4dc9ad2f7db235*)* through the Open Science Framework before analysis. The same link also provides analysis scripts. Simulation analyses indicated that the current sample size and analytic approach provide sufficient statistical power to assess the associations of interest (see the Power analysis section below).

## Participants

Data were available from a parent study to investigate how self-reported PT dimensions were related to later engagement of different ER strategies. The parent study (Garcia, n.d.) was pre-registered (https://osf.io/yc8e6/overview*).* The original sample consisted of 429 college student participants recruited from a private university in the Midwest. The study was posted on SONA, an online platform that advertises research opportunities to university students for course research credit compensation. Participants were required to be at least 18 years old and have access to a device capable of playing the study audio. Five participants were excluded because of this criterion. We also limited our analyses to daily diaries from the PT writing portion that were 20 words or more (the rationale of this threshold is described below). As a result, a total of 378 observations (12.1%) were excluded from the original 3,137 diaries. At the person-level, data from eleven participants were entirely excluded, and data from 115 participants were partially excluded. In the final sample **(***N* = 413; Texts *N* = 2787; *M*_*age*_ = 19.6, *SD*_*age*_ = 1.5; *range*: 18–32**)**, participants’ gender was 52.2% female, 41.6% male, 1.7% non-binary, and 4.5% unknown. Their race/ethnicity was 16.0% Hispanic/Latinx, 38.0% Non-Hispanic White/European American, 26.2% Non-Hispanic Asian, 7.9% Non-Hispanic Black/African American, and 3.8% Non-Hispanic multi-racial. Like other college populations, the current sample showed elevated depressive symptoms (Center for Epidemiologic Studies Depression Scale (Radloff, 1977): Mean = 39.6, *SD* = 15.6, Range: 20–100) and elevated anxiety symptoms (General Anxiety Disorder-7 (Spitzer et al., 2006): Mean = 15.0, *SD* = 5.2, Range: 7–28).

## Procedures

All procedures were approved by the university’s Institutional Review Board. Participants interested in the study were sent an online informed consent form. After consenting to participate, participants received an email the following day at 9:00 pm with a hyperlink to an online baseline session. During the 30-minute baseline session, which served as the first day of the 10-day study procedure, participants received psychoeducation about the definition and common themes of PT and completed an online tutorial and training on their daily tasks. Participants continued to receive a link to their daily tasks at 9:00 pm for the following nine days and were asked to complete them before they went to sleep. Each link remained accessible until 5:00 am the following morning.

Starting with the baseline session, participants completed the following tasks in a set order every day, modified from Krahé et al. (2016): (a) report their momentary affect; (b) write their negative, perseverative thoughts about a recent experience for two minutes on a computer (recommended) or smartphone; (c) spend one minute thinking about said experience; (d) report their self-rated dimensions of momentary PT; (e) report momentary affect; (f) spend one minute attempting to feel less negative about the experience; and (g) report their self-rated dimensions of momentary PT, ER strategy choice and engagement, and momentary affect; (h) watch a short positive video for mood smoothing.

On Day 10, participants were debriefed after completing their daily tasks. They were compensated with research participation credit that was prorated based on the number of daily tasks they completed.

## Measures

### **Momentary ER Strategy Choice**

During their daily tasks, after the ER period, participants rated the extent to which they engaged in six ER strategies from 1 (*Not at all)* to 7 (*A great deal).* Each item was dichotomized to indicate whether a strategy was selected or not, with 1 (*not at all*) categorized as 0 (*not selected*) and 2 to 7 categorized as 1 (*selected*). The six strategies were (a) reappraisal (“Tried to think about something more positively”), (b) problem-solving (“I tried to think about a way to fix the problem”), (c) acceptance (“I tried to accept that this is the way things are”), (d) experiential avoidance (“I tried to get rid of negative thoughts, feelings, or sensations”), (e) expressive suppression (“Tried to not let my feelings show”), (f) distraction (“Tried to think about something else to change how I was feeling [i.e., distract myself]”). Items for reappraisal, suppression, and distraction have been utilized previously by Eldesouky and English (2018). Items for acceptance and problem-solving were administered by Short et al. (2018). The item for experiential avoidance was modeled after Naragon-Gainey et al. (2017). The six ER strategies showed fair to moderate intraclass correlations (ICC; range: 0.26–0.46), suggesting substantial proportions of within-person variance.

### **PT Linguistic Measures**

Repetitiveness was captured through sentence embeddings (Iter et al., 2018), and the other PT dimensions were captured through features identified by Pennebaker’s Linguistic Inquiry and Word Count (LIWC) program (Boyd et al., 2022), with details described in the section below. We included the following PT dimensions: (a) valence, (b) self-focus, (c) interpersonal, (d) discrepancies, (e) situational certainty, (f) temporal orientation, and (g) repetitiveness. Detailed mapping between linguistic features and PT dimensions can be seen in Table 1. Cronbach’s α scores are postulated to underestimate the reliability of linguistic markers, with our chosen markers typically having low to medium scores (α’s = 0.22 − 0.71; Boyd et al., 2022). When calculating Cronbach’s alpha, the frequency of a word was used to indicate the score of a word. As a result, the Cronbach’s alpha will be reduced simply because the frequency of similar words can be mutually exclusive (e.g., when the frequency of “happy” is high, usually they won’t use “joyful,” although including both in the dictionary is reasonable). Therefore, the Kuder-Richardson Formula 20 (KR-20), which rated each word based on their presence or not (1/0), is considered a more accurate measure of the reliability of linguistic markers, with our chosen markers typically having excellent reliability (KR-20s = 0.85-0.99; Boyd et al., 2022). The PT linguistic features showed fair ICC scores within the final sample (range: 0.13–0.27), suggesting substantial proportions of within-person variances.

## Analytic Plan

### Power analysis

Since there was no research on PT dimensions and ER, the parent study ran a pilot study that recruited ten participants, assessed up to nine times each, to gain effect sizes. The estimates of means, variances, and covariances from the pilot data (See Table ST1 in the supplemental materials) were used for simulations in power analysis. Notably, the parent study tested self-report PT dimensions and engagement of ER strategies, and the estimates were calculated as such from linear-mixed effects models. Across simulations, different numbers of measurement occasions (from 5 to 10 measurement points within an individual) and numbers of people (from 5 to 1000 individuals) were tested. Each simulation generated 2000 simulated data sets. Across simulations, the proportion of rejected null hypotheses represented the empirical power rate. Table ST2 in the supplemental materials presents the number of individuals and the number of measurement occasions for associations between each PT dimension and each ER strategy engagement needed. Since different associations of PT/ER require different sample sizes and measurement points, the parent study made decisions to balance feasibility (e.g., collecting less than 1000 individuals) and maximize the number of effects detected. Power analysis results indicated a design with 424 participants and eight repeated assessments, and we decided to collect 10 measurement points and aimed to reach 435 individuals, considering attrition rates and to be conservative. Data collection was conducted from March 2022 to December 2022. The parent study (pre-registered) collected data from 431 (99% of the target goal) participants, but stopped recruitment due to time constraints. All Power analysis codes can be seen in https://osf.io/zu68k/?view_only=93938cf95ded47eeaa4dc9ad2f7db235

### Linguistic Feature Extraction

The minimum threshold of 20 words for further analyses was decided to balance the sample size to be retained, the length of the writing portion, and recommendations in the literature (Kern et al., 2016). For sensitivity analyses, we examined the robustness of our results based on two other sub-datasets as pre-registered: one with a 40-word threshold, and another with a 60-word threshold^1^ . Sensitivity analysis results are presented in the supplemental materials.

The third author reviewed the text and identified special characters that were unidentified in the PT writing responses (e.g., Apostrophes [‘] = ‚Äô, Three dots […] = ‚Ä¶), and they were removed before analysis. We did not use stemming or lemmatization because LIWC, the operationalization method of most of our PT dimensions, was based on calculating frequencies of words in a dictionary. Lemmatization could negatively impact the definition of some LIWC dimensions. After manually cleaning some special characters generated by Qualtrics in the texts, we used the LIWC program (Boyd et al., 2022) to analyze the text of each individual’s PT diaries. LIWC computes the percentage of words that fall within word categories. Language features, including negative tone, first-person singular pronouns, discrepancies, certitude, social processes, past-focus, and future-focus were extracted.

Following research on linguistic models of PT (Stade et al., 2023), repetitiveness scores were extracted through sentence embeddings(Iter et al., 2018). Embedding is a process of giving numerical values to non-numerical objects (e.g., words, sentences, punctuations, images). In sentence embeddings, instead of assigning a single number to a sentence, a vector is usually used to represent a word or sentence. A vector is a combination of more than one number that carries both magnitude and direction information. It can be represented as a directed line segment, where the arrow’s length is the magnitude, and the arrow’s orientation is the direction. For example, [2, -1] is a vector from a two-dimensional space. The cosine of the angle between two vectors could represent how close two vectors are in a dimensional space. Thus, we were able to quantify similarities between two sentences through the cosines between the two vectors that represent the two sentences. First, we divided the original texts into individual sentences through a sentence tokenizer function from the Natural Language Toolkit (Loper & Bird, 2002). Then, each sentence was mapped to a 1024-dimensional vector space using a pre-trained RoBERTa model (Reimers & Gurevych, 2019). Semantic similarity was indicated by proximity between sentence embeddings in the vector space. We then calculated the average scores of all cosine similarities between every pair of sentences as the numerical repetitiveness score of a PT episode.

### Data Analysis

Outliers in the data (over three standard deviations) were Winsorized to the three standard deviation values before analyses. Multilevel logistic regression models (MLMs) were performed in R version 4.4.0. (R Core Team, 2019) to assess how changes in PT dimensions assessed via linguistic features predicted subsequent ER strategy selection. Models included two levels: PT linguistic dimensions (level 1) nested within persons (level 2). We ran a separate MLM for each outcome variable (i.e., the selection or not of the six ER strategies), in which all predictors (i.e., PT linguistic dimensions) and their random intercepts were included simultaneously. We adjusted the alpha level based on Rubin (2021)’s suggestions for alpha adjustment. More specifically, we did not correct for alpha for hypothesis testing in Aim 1, in which each result must be significant to reject each associated individual null hypothesis (i.e., testing associations between one PT dimension and one ER choice, individual testing). We used the conventional alpha-level (0.05) for any association that was used to test a pre-registered hypothesis, but corrected for false detection rates (FDR) based on the number of exploratory tests for exploratory hypotheses. Then, we corrected for FDR for hypothesis testing for Aim 2 and Aim 3, in which at least one test result must be significant to reject the associated joint null hypothesis. Specifically, hypotheses in Aims 2 and 3 examined how PT dimensions will relate to different types of ER choices. We will correct for FDR based on the total number of tests for each of these hypotheses since each requires multiple tests (disjunction testing).

## Results

### Descriptives and Correlations

Descriptive information for the momentary variables (within-person means, within-person standard deviations, ICCs) and bivariate correlations (within- and between) are summarized in Table 3. Within- and between-person correlations between PT dimensions were weak to modest (-0.28 < *r*s_*within*_*<* 0.14, − 0.25 < *r*s_*between*_*<* 0.35). Within-person correlations between ER strategies ranged from 0.01 to 0.16. Problem-solving and reappraisal showed a strong between-person association (*r* = .54, *p* < .001). Three disengagement strategies (avoidance, distraction, suppression) showed strong between-person correlations (*r*s = 0.50, 0.50, 0.50, all *p*s < 0.001). Within-person correlations between linguistic PT dimensions and self-reported PT items revealed moderate associations for self-focus (*r* = .17, *p* < .001), past-focus (*r* = .21, *p* < .001), and future focus (*r* = .21, *p* < .001), strong associations for interpersonal processes (*r* = .49, *p* < .001), and weak or no associations for negative valence (*r* = .02, *p* = .37) and repetitiveness (*r* = .01, *p* = .63). 

**Table 3:** Descriptive statistics, between- and within-person bivariate correlations for primary study variables

	M [SD]	Range	ICC	1	2	3	4	5	6	7	8	9	10	11	12	13	14
*Emotion Regulation Strategy Selection*																	
1. Reappraisal	0.07 [0.17]	0–1	0.33	---	0.07**	0.03	0.14**	0.12**	0.12**	− 0.02	0.01	0.01	− 0.03	− 0.04*	− 0.02	0.02	0.04
2. Expressive Suppression	0.15 [0.22]	0–1	0.46	0.22**	---	0.16**	0.09**	0.03	0.16**	− 0.01	0.03	− 0.01	− 0.01	0.03	0.00	0.02	0.00
3. Distraction	0.13 [0.21]	0–1	0.38	0.06	0.50**	---	0.07**	0.02	0.15**	0.02	0.05**	0.01	0.01	− 0.03	0.03	− 0.02	0.01
4. Acceptance	0.17 [0.23]	0–1	0.29	0.28**	0.35**	0.39**	---	0.01	0.06**	− 0.01	− 0.02	− 0.01	− 0.01	0.00	0.02	0.00	0.00
5. Problem Solving	0.17 [0.23]	0–1	0.26	0.54**	0.36**	0.22**	0.35**	---	0.10**	0.01	0.06**	0.04	− 0.04*	− 0.11**	− 0.06**	0.04*	0.05*
6. Experiential Avoidance	0.06 [0.15]	0–1	0.41	0.17**	0.50**	0.50**	0.31**	0.28**	---	0.02	0.02	0.01	− 0.04	− 0.03	− 0.03	0.03	0.02
*Perseverative Thought Dimensions*																	
7. Negative Valence	2.52 [1.22]	0-7.25	0.27	0.01	0.08	0.08	0.03	− 0.04	0.05	---	0.03	− 0.09**	0.01	0.05*	0.02	− 0.13**	0.13**
8. Self-Focus	3.48 [1.47]	0.08–8.96	0.27	0.03	0.04	− 0.02	0.02	0.00	− 0.01	0.10*	---	0.14**	− 0.02	− 0.28**	− 0.02	0.05**	0.09**
9. Discrepancy	1.93 [0.86]	0-5.12	0.16	− 0.02	− 0.06	− 0.03	0.02	0.00	− 0.05	− 0.06	0.15**	---	− 0.02	− 0.08**	− 0.01	− 0.02	− 0.01
10. Certainty	0.93 [0.61]	0-2.55	0.18	− 0.01	− 0.06	− 0.01	− 0.08	− 0.02	− 0.02	− 0.13**	− 0.13**	− 0.12*	---	0.01	0.00	− 0.04	− 0.01
11. Interpersonal	4.63 [2.03]	0-11.62	0.13	− 0.01	− 0.01	− 0.02	− 0.01	− 0.17**	0.00	0.03	− 0.15**	0.08	0.14**	---	0.05*	− 0.19**	0.02
12. Past Focus	3.05 [1.51]	0-10.77	0.17	− 0.03	− 0.09	− 0.04	0.04	− 0.05	0.00	− 0.07	− 0.12*	− 0.11*	0.09	0.10*	---	− 0.18**	− 0.06**
13. Future Focus	2.28 [1.08]	0-5.87	0.14	0.05	− 0.04	− 0.02	0.05	0.07	− 0.04	0.01	0.08	0.16**	− 0.14**	− 0.14**	− 0.25**	---	0.01
14. Repetitiveness	0.09 [0.04]	0-0.27	0.22	0.14**	0.16**	0.17**	0.13*	0.07	0.11*	0.35**	0.13**	− 0.11*	0.07	0.21**	− 0.11*	− 0.02	---

*M* and *SD*s for momentary measures are average within-person *M* and *SD*s across participants. Between-person correlations are below the diagonal. Within-person associations for momentary measures are above the diagonal. *ICC *intraclass correlation

*p < .05, **p < .01

### Aim 1: PT Dimensions predicting ER Choices

Main findings were presented in Table 4, and detailed findings were presented in Table 2. Consistent with our hypothesis, higher negative tone during PT significantly predicted an increased likelihood of later selecting distraction (*Odds Ratio* [*OR*] = 1.25, *Standard Error of OR *[*SE*] = 0.13, *p* = .032). Consistent with our hypothesis, more first-person singular pronoun usage during PT significantly predicted an increased likelihood of later selecting expressive suppression (*OR* = 1.21, *SE* = 0.13, *p* = .016) and experiential avoidance (*OR* = 1.36, *SE* = 0.21, *p* = .047). Consistent with our hypothesis, higher certitude language during PT (indicating greater situational certainty; *OR* = 0.86, *SE* = 0.06, *p* = .046) and more past focus during PT (*OR* = 0.74, *SE* = 0.06, *p* = .023) predicted a decreased likelihood of later selecting problem solving.

**Table 4:** Main findings between perseverative thoughts dimensions and selection of engagement emotion regulation strategies at the within-individual level

PT Linguistic Dimensions	Within-person *r* with self-report PT item	Significant findings in MLM
1. Negative Valence	0.02	Distraction ↑
2. Self-Focus	0.17***	Experiential Avoidance ↑, Expressive Suppression ↑
3. Discrepancies	no corresponding self-report PT item	ns
4. Situational Certainty	− 0.03	Problem-Solving ↓, Experiential Avoidance ↓
5. Interpersonal	0.49***	Problem-Solving ↓, Expressive Suppression ↑ (F), Reappraisal ↓ (F)
6. Past Focus	0.21***	Problem-Solving ↓
7. Future Focus	0.21***	Experiential Avoidance ↑ (F)
8. Repetitiveness	0.01	Reappraisal ↑

*MLM *multilevel logistic models. *PT *perseverative thoughts. (F) denotes significant associations that did not survive False Detection Rates (FDR) corrections. ns = no significant findings

***p < .001

Exploratory tests indicated that more words on social processes during PT significantly predicted a decreased likelihood of later selecting problem-solving (*OR* = 0.66, *SE* = 0.06, *p* < .001). There are some trends in associations of experiential avoidance with less certitude language (*OR* = 0.76, *SE* = 0.10, *p* = .040) and more future focus (*OR* = 1.49, *SE* = 0.20, *p* = .020), and association of social processes during PT with bigger likelihood of selecting expressive suppression (*OR* = 1.23, *SE* = 0.11, *p* = .018), and a lower likelihood of selecting reappraisal (*OR* = 0.74, *SE* = 0.09, *p* = .019), although they did not survive FDR corrections. Contrary to our hypotheses, a higher level of repetitiveness during PT predicted a greater likelihood of later selecting reappraisal (*OR* = 1.37, *SE* = 0.19, *p* = .046). 

### Aim 2–3: PT Dimensions and ER Choice in Big Categories

Consistent with our hypothesis, after FDR corrections, results showed that greater use of interpersonal words during PT was associated with less subsequent choice to engage with their emotions. There was no strong evidence to reject our null hypotheses that a PT dimension predicteda higher likelihood of choosing to disengage from emotions. Overall, we could reject the null hypothesis that thought content from PT was not a contextual factor that predicted subsequent ER choices.

## Discussion

The current study utilized a 10-day intensive longitudinal design and novel experimental PT induction in conjunction with computational linguistics to examine how dimensions of PT predicted subsequent ER strategy selection within individuals. Given research highlighting the importance of ER *selection*, rather than ER implementation, in the emotion dysregulation that characterizes MDD (Liu & Thompson, 2017), identifying how specific dimensions of PT are linked with subsequent ER strategy choice represents an important step towards identifying targetable mechanisms by which PT may lead to emotion dysregulation. Further, our objective assessment of PT dimensions through written language allowed us to assess dimensions that are difficult to measure using self-report (e.g., repetitiveness).

Although researchers have typically conceptualized contexts of ER strategy choice as emotional (i.e., emotion intensity, type of emotion; e.g., Aldao & Nolen-Hoeksema, 2012), social (i.e., social circumstances; e.g., English et al., 2017), or motivational(e.g., Tamir et al., 2020), our study proposed PT as a cognitive contextual feature that may influence subsequent selection of ER strategies, an idea that was further supported by the data (Aim 3 supported after FDR correction). As a process that is usually involuntary to start and difficult to end, PT is not only a response to emotions, but can be an ongoing context that influences how individuals respond to emotions. There are several potential mechanisms by which PT may influence ER strategy choice, including PT’s influence on cognitive resources, attention control and bias, or cognitive flexibility (Whitmer & Gotlib, 2013). Direct investigation of how PT influences ER strategy choice through these links is worth testing in future research. Additionally, like others who have viewed PT as a failure in cognitive control (Singh et al., 2025; Whitmer & Gotlib, 2013; Zetsche et al., 2018), we have interpreted the present results through the lens of PT as a cognitive context of ER, but we argue that our results are also valuable even for readers who holds different conceptualization of PT. For example, readers who view PT as an ER strategy could interpret our results as poly-regulation in a sample vulnerable to internalizing psychopathology and generate hypotheses from those interpretations.

Several hypotheses about the ways in which PT dimensions might shape individuals’ tendencies to disengage from or engage with emotional experiences (Aim 2) were not consistently supported by the data, possibly because the associations between PT dimensions and ER choice may be more complicated (Aim 1 hypotheses). As hypothesized, after engaging in more negatively valenced PT, individuals were more likely to distract themselves, consistent with research that distraction is the only elevated ER strategy choice among individuals with current MDD compared to healthy controls (Liu et al., 2023). As hypothesized, when individuals focused more on themselves during PT, they were subsequently more likely to avoid their internal experiences and suppress their expressions. This may provide insights into how social worry may influence on ER choice to develop and maintain social phobia, given the central role of self-focused cognition in models of social phobia (Clark et al., 2005; Rapee & Heimberg, 1997) and that expressive suppression reduced rapport and inhibited relationship formation (Butler et al., 2003). Finally, as expected, higher situation certainty, more social words, and more past focus during PT predicted less problem solving, possibly because problems in those situations were perceived as less malleable. Our findings on specific PT dimensions and ER strategy choice were largely consistent with existing theories and evidence, and they further contributed new mechanistic insights into these processes.

Inconsistent with the hypotheses, a high level of certitude predicted a lower likelihood of selecting experiential avoidance, and a higher level of repetitiveness during PT subsequently predicted a greater likelihood of selecting reappraisal. These could be due to a measurement issue, as reflected by the low within-person correlations between self-rated certitude and repetitiveness in PT and their linguistic measures. Words like “really” could reflect more suspicion. The sentence-to-sentence similarity score may have overestimated repetitiveness in cases where sentence structure is similar, but the actual content is distinct. Alternatively, repetitiveness during PT being associated with subsequently higher reappraisal might be meaningful, as higher self-reported repetitiveness also predicted higher ratings of reappraisal in the same dataset (Garcia, n.d.). The conceptualization of PT started from the distinction between maladaptive rumination (i.e., brooding) and adaptive reflective thinking (i.e., intellectual self-reflection; Nolen-Hoeksema et al., 2008), although their relations have rarely been investigated within-person. Our results suggest that reappraisal may be more easily initiated following successful disengagement from PT, as our study procedures instructed participants to disengage from PT before ER. In daily life, these two processes may happen simultaneously as PT is hard to disengage. Future research could benefit from further uncovering the dynamic associations between PT and ER choices (or other ER processes) with even higher ecological validity.

To interpret the relative magnitude of our findings, we consider the effect sizes of similar associations within the linguistic-psychological literature. In a study on language and personality traits (*N* = 69,792), none of the linguistic correlates of Big-5 personality traits yielded |r| over 0.19 (Kern et al., 2014). The link between first-person singular pronoun use and depression is arguably one of the more robust effects(Holtzman, 2017), with an overall effect of *r* = .13. Within these contexts, the correlations (see Table 3) between PT linguistic dimensions and ER strategy selection, though weak to moderate, are equal to or larger than typical correlations found in linguistic psychology research, indicating they likely represent meaningful insights.

The current study also had limitations worth noting. Firstly, the use of a college sample limits the generalizability of our findings. For example, the within-person associations between repetitiveness and reappraisal may be negative in samples with different clinical manifestations of emotion dysregulation. Secondly, the act of writing may alter the process of PT. Less intrusive methods, such as interviews or passive audio recordings, are promising methods to increase the ecological validity of linguistic feature extraction. Thirdly, the current study is limited by not assessing interpersonal context when we measured PT content. Future research could benefit from investigating how PT interacts with other contexts to influence ER choice, for example, social contexts (e.g., being alone or with others, being with close versus non-close others, or being with the person who stressed them). Fourthly, most of the PT dimensions were assessed via LIWC, a dictionary-based tool, which did not consider how word meanings may change based on contexts around the words (e.g., “really” with a single score in LIWC certitude can mean differently across contexts). More recent advances in natural language processing (embedding-based approaches) and generative artificial intelligence open new avenues for linguistic measures of PT dimensions. Future research could benefit from testing better linguistic measures of PT dimensions, especially for those that showed weak correlations with self-report PT dimensions in the current data (i.e., certitude, negative valence, and repetitiveness). Finally, our work focused solely on ER strategy choice, and further research is warranted to investigate how other aspects of the ER process may differ in the context of PT (e.g., ER implementation).

The current research represents a crucial first step towards understanding how dimensions of PT, as assessed through written language, are differentially linked with subsequent ER strategy selection using an intensive longitudinal design and experimental PT-induction paradigm. Negative valence predicted a higher likelihood of distraction. Self-focus predicted a higher likelihood of experiential avoidance and expressive suppression. Situational certainty, interpersonal words, and past focus predicted a lower likelihood of problem solving. Overall, evidence supports PT dimensions as contextual factors to influence ER choice, and highlights the promise of computational linguistics for advancing the understanding of dynamic, covert, and multidimensional processes like PT.

## Supplementary Information

Below is the link to the electronic supplementary material.

Supplementary Material 1

## Footnotes

1 Fromthe additional linear mixed effects models that we conducted, depressivesymptoms did not predict word count, neither in the total sample (*p* =.72) nor in the analytic sample (*p* = 0.41).

## Additional Information

### Data Availability

The data that support the findings of this study are available from the corresponding author upon reasonable request.

### Code Availability

The analysis code can be found at https://osf.io/skmh6/?view_only=9ac57b830f4849ae9f885a25d326bfa7.

## References

[CR1] Aldao, A., & Nolen-Hoeksema, S. (2012). The influence of context on the implementation of adaptive emotion regulation strategies. *Behaviour Research and Therapy*, *50*(7–8), 493–501. 10.1016/j.brat.2012.04.004 22659159 10.1016/j.brat.2012.04.004

[CR2] Basterfield, C., Fitzsimmons-Craft, E. E., Taylor, C. B., Eisenberg, D., Wilfley, D. E., & Newman, M. G. (2024). Internalizing psychopathology and its links to suicidal ideation, dysfunctional attitudes, and help-seeking readiness in a National sample of college students. *Journal of Affective Disorders*, *350*, 255–263. 10.1016/j.jad.2024.01.058 38224742 10.1016/j.jad.2024.01.058 PMC11057016

[CR3] Boyd, R. L., Ashokkumar, A., Seraj, S., & Pennebaker, J. W. (2022). *The development and psychometric properties of LIWC-22.* University of Texas at Austin, p 10. https://www.liwc.app

[CR4] Brockmeyer, T., Zimmermann, J., Kulessa, D., Hautzinger, M., Bents, H., Friederich, H. C., Herzog, W., & Backenstrass, M. (2015). Me, myself, and I: Self-referent word use as an indicator of self-focused attention in relation to depression and anxiety. *Frontiers in Psychology*, *6*, 1564. 10.3389/fpsyg.2015.01564 26500601 10.3389/fpsyg.2015.01564 PMC4598574

[CR5] Butler, E. A., Egloff, B., Wlhelm, F. H., Smith, N. C., Erickson, E. A., & Gross, J. J. (2003). The social consequences of expressive suppression. *Emotion*, *3*(1), 48–67. 10.1037/1528-3542.3.1.48 12899316 10.1037/1528-3542.3.1.48

[CR6] Clark, D. M., Crozier, W. R., & Alden, L. E. (2005). A cognitive perspective on social phobia. In W. R. Crozier & L. E. Alden (Eds.), *The essential handbook of social anxiety for clinicians *(pp. 193–218). John Wiley & Sons, Ltd

[CR7] Corbin, L., Griner, E., Seyedi, S., Jiang, Z., Roberts, K., Boazak, M., Rad, A. B., Clifford, G. D., & Cotes, R. O. (2023). A comparison of linguistic patterns between individuals with current major depressive disorder, past major depressive disorder, and controls in a virtual, psychiatric research interview. *Journal of Affective Disorders Reports*, *14*, 100645. 10.1016/j.jadr.2023.100645

[CR8] Davey, G. C., Meeten, F., & Field, A. P. (2022). What’s worrying our students? Increasing worry levels over two decades and a new measure of student worry frequency and domains. *Cognitive Therapy and Research*, 1–14. 10.1007/s10608-021-10270-0 10.1007/s10608-021-10270-0 PMC8501938 34658461

[CR9] Ehring, T., & Watkins, E. R. (2008). Repetitive negative thinking as a transdiagnostic process. *International Journal of Cognitive Therapy*, *1*(3), 192–205. 10.1521/ijct.2008.1.3.192

[CR51] Eldesouky, L., & English, T. (2018). Another year older, another year wiser? Emotion regulation strategy selection and flexibility across adulthood. *Psychology and Aging, 33*(4), 572–585. 10.1037/pag0000251 10.1037/pag0000251 PMC6002946 29745687

[CR10] English, T., Lee, I. A., John, O. P., & Gross, J. J. (2017). Emotion regulation strategy selection in daily life: The role of social context and goals. *Motivation and Emotion*, *41*, 230–242. 10.1007/s11031-016-9597-z 28652647 10.1007/s11031-016-9597-z PMC5482525

[CR11] Fisher, A. J., Medaglia, J. D., & Jeronimus, B. F. (2018). Lack of group-to-individual generalizability is a threat to human subjects research. *Proceedings of the National Academy of Sciences*, *115*(27), E6106–E6115. 10.1073/pnas.1711978115 10.1073/pnas.1711978115 PMC6142277 29915059

[CR12] Garcia, B. (n.d.). *Emotion regulation flexibility: Investigating perseverative thinking as a novel context*. 10.7936/ha3p-3m53

[CR45] Gross, J. J. (2015). The extended process model of emotion regulation: Elaborations, applications, and future directions. *Psychological Inquiry, 26*(1), 130–137. 10.1080/1047840X.2015.989751

[CR13] Hallion, L. S., Wright, A. G., Joormann, J., Kusmierski, S. N., Coutanche, M. N., & Caulfield, M. K. (2022). A five-factor model of perseverative thought. *Journal of Psychopathology and Clinical Science*, *131*(3), 235. 10.1037/abn0000737 35230863 10.1037/abn0000737 PMC9439587

[CR14] Hankin, B. L., Snyder, H. R., Gulley, L. D., Schweizer, T. H., Bijttebier, P., Nelis, S., Toh, G., & Vasey, M. W. (2016). Understanding comorbidity among internalizing problems: Integrating latent structural models of psychopathology and risk mechanisms. *Development and Psychopathology*, *28*(4pt1), 987–1012. 10.1017/S0954579416000663 27739389 10.1017/S0954579416000663 PMC5119897

[CR15] Holtzman, N. S. (2017). A meta-analysis of correlations between depression and first person singular pronoun use. *Journal of Research in Personality*, *68*, 63–68. 10.1016/j.jrp.2017.02.005

[CR16] Hong, R. Y. (2007). Worry and rumination: Differential associations with anxious and depressive symptoms and coping behavior. *Behaviour Research and Therapy*, *45*(2), 277–290. 10.1016/j.brat.2006.03.006 16635479 10.1016/j.brat.2006.03.006

[CR17] Iter, D., Yoon, J., & Jurafsky, D. (2018). *Automatic detection of incoherent speech for diagnosing schizophrenia,* pp 136–146. 10.18653/v1/W18-0615

[CR18] Kern, M. L., Eichstaedt, J. C., Schwartz, H. A., Dziurzynski, L., Ungar, L. H., Stillwell, D. J., Kosinski, M., Ramones, S. M., & Seligman, M. E. (2014). The online social self: An open vocabulary approach to personality. *Assessment*, *21*(2), 158–169. 10.1177/1073191113514104 24322010 10.1177/1073191113514104

[CR19] Kern, M. L., Park, G., Eichstaedt, J. C., Schwartz, H. A., Sap, M., Smith, L. K., & Ungar, L. H. (2016). Gaining insights from social media language: Methodologies and challenges. *Psychological Methods*, *21*(4), 507. 10.1037/met0000091 27505683 10.1037/met0000091

[CR20] Kircanski, K., Thompson, R. J., Sorenson, J., Sherdell, L., & Gotlib, I. H. (2018). The everyday dynamics of rumination and worry: Precipitant events and affective consequences. *Cognition and Emotion*, *32*(7), 1424–1436. 10.1080/02699931.2017.1278679 28103761 10.1080/02699931.2017.1278679 PMC6218316

[CR50] Koster, E. H., De Lissnyder, E., Derakshan, N., & De Raedt, R. (2011). Understanding depressive rumination from a cognitive science perspective: The impaired disengagement hypothesis. *Clinical Psychology Review, 31*(1), 138–145. 10.1016/j.cpr.2010.08.005 10.1016/j.cpr.2010.08.005 20817334

[CR21] Krahé, C., Mathews, A., Whyte, J., & Hirsch, C. R. (2016). Cognitive bias modification for interpretation with and without prior repetitive negative thinking to reduce worry and rumination in generalised anxiety disorder and depression: Protocol for a multisession experimental study with an active control condition. *British Medical Journal Open*, *6*(12), e013404. 10.1136/bmjopen-2016-013404 10.1136/bmjopen-2016-013404 PMC5168672 27986741

[CR22] Liu, D. Y., Springstein, T., Tuck, A. B., English, T., & Thompson, R. J. (2023). Everyday emotion regulation goals, motives, and strategies in current and remitted major depressive disorder: An experience sampling study. *Journal of Psychopathology and Clinical Science*, *132*(5), 594. 10.1037/abn0000831 37199995 10.1037/abn0000831

[CR23] Liu, D. Y., & Thompson, R. J. (2017). Selection and implementation of emotion regulation strategies in major depressive disorder: An integrative review. *Clinical Psychology Review*, *57*, 183–194. 10.1016/j.cpr.2017.07.004 28739277 10.1016/j.cpr.2017.07.004

[CR24] Loper, E. & Bird, S. (2002). Nltk: The natural Language toolkit. *ArXiv Preprint Cs/0205028*10.48550/arXiv.cs/0205028.

[CR25] McLaughlin, K. A., & Sheridan, M. A. (2016). Beyond cumulative risk: A dimensional approach to childhood adversity. *Current Directions in Psychological Science*, *25*(4), 239–245. 10.1177/0963721416655883 27773969 10.1177/0963721416655883 PMC5070918

[CR26] McMahon, T. P., & Naragon-Gainey, K. (2019). The multilevel structure of daily emotion-regulation-strategy use: An examination of within-and between-person associations in naturalistic settings. *Clinical Psychological Science*, *7*(2), 321–339. 10.1177/2167702618807408

[CR27] Moberly, N. J., & Watkins, E. R. (2008). Ruminative self-focus, negative life events, and negative affect. *Behaviour Research and Therapy*, *46*(9), 1034–1039. 10.1016/j.brat.2008.06.004 18684437 10.1016/j.brat.2008.06.004 PMC2682175

[CR28] Naragon-Gainey, K., McMahon, T. P., & Chacko, T. P. (2017). The structure of common emotion regulation strategies: A meta-analytic examination. *Psychological Bulletin*, *143*(4), 384. 10.1037/bul0000093 28301202 10.1037/bul0000093

[CR29] Nolen-Hoeksema, S., Wisco, B. E., & Lyubomirsky, S. (2008). Rethinking rumination. *Perspectives on Psychological Science*, *3*(5), 400–424. 10.1111/j.1745-6924.2008.00088.x 26158958 10.1111/j.1745-6924.2008.00088.x

[CR30] Pennebaker, J. W., & Stone, L. D. (2003). Words of wisdom: Language use over the life span. *Journal of Personality and Social Psychology*, *85*(2), 291. 10.1037/0022-3514.85.2.291 12916571 10.1037/0022-3514.85.2.291

[CR31] Radloff, L. S. (1977). The CES-D scale: A self-report depression scale for research in the general population. *Applied Psychological Measurement*, *1*(3), 385–401.

[CR32] Rapee, R. M., & Heimberg, R. G. (1997). A cognitive-behavioral model of anxiety in social phobia. *Behaviour Research and Therapy*, *35*(8), 741–756. 10.1016/S0005-7967(97)00022-3 9256517 10.1016/s0005-7967(97)00022-3

[CR33] Reimers, N., & Gurevych, I. (2019). Sentence-bert: Sentence embeddings using siamese bert-networks. arXiv Preprint arXiv:1908.10084. 10.48550/arXiv.1908.10084

[CR34] Rubin, M. (2021). When to adjust alpha during multiple testing: A consideration of disjunction, conjunction, and individual testing. *Synthese*, *199*, 10969–11000. 10.1007/s11229-021-03276-4

[CR48] Ruscio, A. M., Seitchik, A. E., Gentes, E. L., Jones, J. D., & Hallion, L. S. (2011). Perseverative thought: A robust predictor of response to emotional challenge in generalized anxiety disorder and major depressive disorder. *Behaviour Research and Therapy, 49*(12), 867–874. 10.1016/j.brat.2011.10.001 10.1016/j.brat.2011.10.001 PMC3514044 22030295

[CR35] Segerstrom, S. C., Hardy, J. K., Evans, D. R., Boggero, I. A., Alden, L. E., & Stanton, A. L. (2016). Briefly assessing repetitive thought dimensions: Valence, purpose, and total. *Assessment*, *23*(5), 614–623. 10.1177/1073191115586458 26019299 10.1177/1073191115586458 PMC4766040

[CR47] Sheppes, G., Scheibe, S., Suri, G., & Gross, J. J. (2011). Emotion-regulation choice. *Psychological Science, 22*(11), 1391–1396. 10.1177/0956797611418350 10.1177/0956797611418350 21960251

[CR46] Sheppes, G., Scheibe, S., Suri, G., Radu, P., Blechert, J., & Gross, J. J. (2014). Emotion regulation choice: a conceptual framework and supporting evidence. *Journal of Experimental Psychology: General, 143*(1), 163. 10.1037/a0030831 10.1037/a0030831 23163767

[CR52] Short, N. A., Boffa, J. W., Clancy, K., & Schmidt, N. B. (2018). Effects of emotion regulation strategy use in response to stressors on PTSD symptoms: An ecological momentary assessment study.* Journal of Affective Disorders, 230*, 77–83. 10.1016/j.jad.2017.12.063 10.1016/j.jad.2017.12.063 PMC5811367 29407542

[CR36] Singh, S., Li, B., Gerhard, S., Nunes, A., & Becker, S. (2025). Emotional modulation of inhibitory control in rumination from empirical and computational perspectives. *Cognitive Affective & Behavioral Neuroscience*, 1–25. 10.3758/s13415-025-01360-7 10.3758/s13415-025-01360-7 PMC12847145 41168557

[CR37] Spinhoven, P., Drost, J., van Hemert, B., & Penninx, B. W. (2015). Common rather than unique aspects of repetitive negative thinking are related to depressive and anxiety disorders and symptoms. *Journal of Anxiety Disorders*, *33*, 45–52. 10.1016/j.janxdis.2015.05.001 26004746 10.1016/j.janxdis.2015.05.001

[CR38] Spitzer, R. L., Kroenke, K., Williams, J. B., & Löwe, B. (2006). A brief measure for assessing generalized anxiety disorder: The GAD-7. *Archives of Internal Medicine*, *166*(10), 1092–1097. 10.1001/archinte.166.10.1092 16717171 10.1001/archinte.166.10.1092

[CR39] Stade, E. C., Ungar, L., Havaldar, S., & Ruscio, A. M. (2023). Perseverative thinking is associated with features of spoken Language. *Behaviour Research and Therapy*, *165*, 104307. 10.1016/j.brat.2023.104307 37121016 10.1016/j.brat.2023.104307 PMC10263193

[CR40] Szkodny, L. E., & Newman, M. G. (2019). Delineating characteristics of maladaptive repetitive thought: Development and preliminary validation of the perseverative cognitions questionnaire. *Assessment*, *26*(6), 1084–1104. 10.1177/1073191117698753 28355881 10.1177/1073191117698753 PMC6707362

[CR41] Tamir, M., Vishkin, A., & Gutentag, T. (2020). Emotion regulation is motivated. *Emotion*, *20*(1), 115. 10.1037/emo0000635.supp 31961189 10.1037/emo0000635

[CR42] Vine, V. (2023). A strategy by any other name? Reconsidering the language of emotion regulation. *Clinical Psychology: Science and Practice*, *30*(4), 507–510. 10.1037/cps0000190

[CR49] Watkins, E. R., & Roberts, H. (2020). Reflecting on rumination: Consequences, causes, mechanisms and treatment of rumination. *Behaviour Research and Therapy, 127*, 103573. 10.1016/j.brat.2020.103573 10.1016/j.brat.2020.103573 32087393

[CR43] Whitmer, A. J., & Gotlib, I. H. (2013). An attentional scope model of rumination. *Psychological Bulletin*, *139*(5), 1036–1061. 10.1037/a0030923 23244316 10.1037/a0030923 PMC3773498

[CR44] Zetsche, U., Bürkner, P. C., & Schulze, L. (2018). Shedding light on the association between repetitive negative thinking and deficits in cognitive control–A meta-analysis. *Clinical Psychology Review*, *63*, 56–65. 10.1016/j.cpr.2018.06.001 29913351 10.1016/j.cpr.2018.06.001

