# Supplementary material for: Language of Perseverative Thoughts Predicts Emotion Regulation Strategy Choice
Source: Affect Sci. 2026 Mar 7;7(2):253–64. doi: 10.1007/s42761-026-00357-w (PMC13269565; doi:10.1007/s42761-026-00357-w)
Supplement: Supplementary file 1 — Supplementary Material 1 [file 42761_2026_357_MOESM1_ESM.docx]

Manuscript Submitted to Affective Science

**Language of Perseverative Thoughts Predicts Emotion Regulation Strategy Choice**

Yiqin Zhu^1*^, Kevin Trent^1^, Beatris Garcia^1^, & Renee J. Thompson^1^

^1^Department of Psychological and Brain Sciences, Washington University in St. Louis, St Louis, MO

**Author Note**:

   Yiqin Zhu <https://orcid.org/0000-0002-9456-2375>

Kevin Trent <https://orcid.org/0009-0006-7126-1728>

Renee J. Thompson<https://orcid.org/0000-0002-4479-096X>

*Correspondence concerning this article should be addressed to Yiqin Zhu, Department of Psychological and Brain Sciences, Washington University in St. Louis, 1 Brookings Drive, CB 1125, St. Louis, MO 63130, United States. Email: [yiqin@wustl.edu](mailto:yiqin@wustl.edu)

**Supplemental Materials Table S1.**

*Effect sizes of Perseverative Thoughts Dimensions and Engagement of Emotion Regulation Strategies detected in linear-mixed models Using Data from a Pilot Study (N = 10)*

|  | Cognitive Reappraisal | Expressive Suppression | Distraction | Acceptance | Problem Solve | Experiential Avoidance |
| --- | --- | --- | --- | --- | --- | --- |
| PT Pleasantness | 0.0185 | 0.4989 | -0.1655 | 0.1989 | 0.2284 | 0.2720 |
| PT Repetitiveness | 0.0033 | 0.0140 | 0.0054 | 0.0085 | 0.0018 | 0.0224 |
| PT Certainty | -0.0006 | 0.0032 | 0.0085 | 0.0400 | 0.0027 | 0.0031 |
| PT Controllability | 0.0039 | 0.0054 | 0.0052 | -0.0181 | 0.0293 | 0.0013 |
| PT Temporal Orientation | -0.0754 | 0.2163 | -0.0217 | 0.2906 | 0.2253 | 0.1297 |
| PT Self Focus | -0.0138 | 0.0093 | -0.3198 | 0.5665 | 0.5097 | 0.6304 |
| PT Other Focus | -0.3311 | -0.3258 | -0.3163 | 0.8492 | -0.1655 | -0.3173 |

**Supplemental Materials Table S2.**

*N of People Needed To Achieve Power of .80 for each association as a function of Occasions per Person*

|  | PT Pleasantness | PT Repetitiveness | PT Certainty | PT Controllability | PT Temporal Orientation | PT Self Focus | PT Other Focus |
| --- | --- | --- | --- | --- | --- | --- | --- |
| Occasions Per Person | Cognitive Reappraisal |  |  |  |  |  |  |
| 5 | >1000 | 482 | >1000 | 286 | 337 | >1000 | 85 |
| 6 | >1000 | 407 | >1000 | 273 | 309 | >1000 | 72 |
| 7 | >1000 | 369 | >1000 | 226 | 278 | >1000 | 63 |
| 8 | >1000 | 319 | >1000 | 201 | 249 | >1000 | 55 |
| 9 | >1000 | 288 | >1000 | 178 | 224 | >1000 | 48 |
| 10 | >1000 | 271 | >1000 | 157 | 209 | >1000 | 44 |
| Occasions Per Person | Expressive Suppression |  |  |  |  |  |  |
| 5 | 38 | 75 | 775 | 302 | 111 | >1000 | 182 |
| 6 | 32 | 63 | 658 | 354 | 90 | >1000 | 156 |
| 7 | 27 | 55 | 538 | 239 | 80 | >1000 | 131 |
| 8 | 23 | 47 | 628 | 262 | 70 | >1000 | 117 |
| 9 | 21 | 42 | 448 | 196 | 64 | >1000 | 103 |
| 10 | 19 | 38 | 491 | 178 | 57 | >1000 | 94 |
| Occasions Per Person | Distraction |  |  |  |  |  |  |
| 5 | >1000 | 416 | 146 | 371 | >1000 | 215 | 200 |
| 6 | 277 | 338 | 121 | 625 | >1000 | 206 | 175 |
| 7 | 239 | 298 | 108 | 259 | 988 | 181 | 150 |
| 8 | 214 | 346 | 90 | 238 | >1000 | 159 | 131 |
| 9 | 191 | 227 | 83 | 218 | >1000 | 139 | 119 |
| 10 | 180 | >1000 | 73 | 210 | >1000 | 125 | 110 |
| Occasions Per Person | Acceptance |  |  |  |  |  |  |
| 5 | 220 | 275 | 7 | 37 | 69 | 80 | 31 |
| 6 | 194 | 181 | 181 | 31 | 58 | 68 | 26 |
| 7 | 162 | 156 | 156 | 27 | 49 | 57 | 23 |
| 8 | 144 | 136 | 136 | 24 | 42 | 50 | 20 |
| 9 | 126 | 122 | 122 | 22 | 39 | 42 | 18 |
| 10 | 116 | 108 | 108 | 19 | 36 | 40 | 17 |
| Occasions Per Person | Problem Solve |  |  |  |  |  |  |
| 5 | 203 | >1000 | >1000 | 17 | 120 | 112 | >1000 |
| 6 | 171 | >1000 | 996 | 16 | 103 | 94 | 529 |
| 7 | 151 | >1000 | 893 | 14 | 88 | 83 | >1000 |
| 8 | 129 | >1000 | 818 | 12 | 79 | 71 | 424 |
| 9 | 121 | >1000 | 580 | 10 | 69 | 63 | 367 |
| 10 | 102 | >1000 | 619 | 9 | 64 | 56 | 344 |
| Occasions Per Person | Experiential Avoidance |  |  |  |  |  |  |
| 5 | 74 | 20 | 501 | 584 | 188 | 39 | 120 |
| 6 | 63 | 17 | 432 | >1000 | 158 | 31 | 100 |
| 7 | 55 | 16 | 290 | 817 | 134 | 29 | 85 |
| 8 | 48 | 14 | 322 | >1000 | 119 | 25 | 74 |
| 9 | 42 | 12 | 392 | >1000 | 108 | 23 | 66 |
| 10 | 39 | 11 | 273 | >1000 | 98 | 20 | 62 |

**Supplemental Materials Table S3.**

*Associations between Perseverative Thoughts Dimensions and Selection of Engagement Emotion Regulation Strategies at the Within-Individual Level (texts shorter than 40 words were removed)*

| Disengagement Strategies | Experiential Avoidance | | | | | Expressive Suppression | | | | | Distraction | | | | |
| --- | --- | --- | --- | --- | --- | --- | --- | --- | --- | --- | --- | --- | --- | --- | --- |
|  | **Hyp** | OR | SE | 95% CI | *p* | **Hyp** | OR | SE | 95% CI | *p* | **Hyp** | OR | SE | 95% CI | *p* |
| 1. Negative Valence | **+ (>1)** | 1.42 | 0.31 | [0.93, 2.18] | .11 | **+ (>1)** | 1.13 | 0.12 | [0.92, 1.38] | .25 | **+ (>1)** | 1.23 | 0.15 | [0.97, 1.55] | .08 |
| 2. Self-Focus | **+ (>1)** | **1.50** | **0.29** | **[1.02, 2.21]** | **.04** | **+ (>1)** | 1.28 | 0.13 | [1.04, 1.57] | **.02** | **+ (>1)** | 1.13 | 0.13 | [0.91, 1.41] | .28 |
| 3. Discrepancies | **+ (>1)** | 1.07 | 0.18 | [0.77, 1.50] | .68 | **+ (>1)** | 0.90 | 0.08 | [0.75, 1.07] | .23 | **+ (>1)** | 0.95 | 0.10 | [0.77, 1.17] | .63 |
| 4. Certitude | **E** | 0.88 | 0.15 | [0.63, 1.22] | .44 | **E** | 1.03 | 0.10 | [0.85, 1.24] | .78 | **E** | 1.24 | 0.13 | [1.00, 1.53] | .047 |
| 5. Interpersonal | **E** | 1.03 | 0.18 | [0.73, 1.47] | .86 | **E** | 1.26 | 0.13 | [1.03, 1.54] | **.03** | **E** | 0.90 | 0.10 | [0.73, 1.12] | .35 |
| 6. Past Focus | **+ (>1)** | 0.97 | 0.16 | [0.70, 1.34] | .84 | **+ (>1)** | 0.99 | 0.09 | [0.83, 1.20] | .95 | **+ (>1)** | 1.17 | 0.12 | [0.95, 1.44] | .13 |
| 7. Future Focus | **E** | **1.50** | **0.29** | **[1.02, 2.21]** | **.04** | **E** | 1.19 | 0.12 | [0.98, 1.44] | .09 | **E** | 0.93 | 0.10 | [0.76, 1.15] | .51 |
| 8. Repetitiveness | **+ (>1)** | 1.07 | 0.21 | [0.73, 1.57] | .72 | **+ (>1)** | 1.04 | 0.11 | [0.85, 1.27] | .69 | **+ (>1)** | 1.09 | 0.12 | [0.88, 1.35] | .42 |
| Engagement Strategies | Reappraisal | | | | | Problem solving | | | | | Acceptance | | | | |
|  | **Hyp** | OR | SE | 95% CI | *p* | **Hyp** | OR | SE | 95% CI | *p* | **Hyp** | OR | SE | 95% CI | *p* |
| 1. Negative Valence | **– (<1)** | 0.77 | 0.12 | [0.56, 1.06] | .11 | **– (<1)** | 1.08 | 0.10 | [0.90, 1.29] | .43 | **– (<1)** | 0.92 | 0.08 | [0.77, 1.09] | .33 |
| 2. Self-Focus | **– (<1)** | 0.98 | 0.17 | [0.70, 1.37] | .92 | **– (<1)** | 1.03 | 0.09 | [0.86, 1.23] | .75 | **– (<1)** | 0.91 | 0.09 | [0.76, 1.10] | .32 |
| 3. Discrepancies | **– (<1)** | 1.14 | 0.19 | [0.82, 1.58] | .43 | **– (<1)** | 0.99 | 0.09 | [0.83, 1.17] | .90 | **– (<1)** | 1.01 | 0.09 | [0.85, 1.20] | .93 |
| 4. Certitude | **+ (>1)** | 0.95 | 0.14 | [0.71, 1.28] | .74 | **– (<1)** | 0.83 | 0.07 | [0.70, 0.97] | .02 | **+ (>1)** | 0.95 | 0.08 | [0.80, 1.12] | .51 |
| 5. Interpersonal | **E** | 0.82 | 0.13 | [0.61, 1.11] | .20 | **E** | 0.66 | 0.06 | [0.56, 0.78] | **<.001** | **E** | 1.00 | 0.09 | [0.84, 1.19] | .98 |
| 6. Past Focus | **+ (>1)** | 0.82 | 0.12 | [0.62, 1.08] | .16 | **– (<1)** | 0.83 | 0.07 | [0.70, 0.98] | **.02** | **+ (>1)** | 1.13 | 0.10 | [0.95, 1.35] | .17 |
| 7. Future Focus | **– (<1)** | 1.06 | 0.18 | [0.76, 1.48] | .71 | **+ (>1)** | 1.08 | 0.10 | [0.89, 1.30] | .43 | **– (<1)** | 1.03 | 0.09 | [0.86, 1.23] | .73 |
| 8. Repetitiveness | **– (<1)** | **1.46** | **0.23** | **[1.07, 1.99]** | **.02** | **– (<1)** | 1.13 | 0.10 | [0.95, 1.35] | .18 | **– (<1)** | 1.08 | 0.10 | [0.91, 1.29] | .37 |

*Note*. “+ (>1)” denotes significant positive associations where OR is larger than 1 in hypotheses, “– (<1)” denotes significant negative associations where OR is less than 1 in hypotheses, “E” denotes Exploratory in hypotheses.

Hyp = Hypothesis OR = Odds Ratio. SE = Standard Error of Odds Ratio. 95% CI = 95% Confidence Interval of Odds Ratio; Coefficients were calculated based on standardized data.

**Supplemental Materials Table S4.**

*Associations between Perseverative Thoughts Dimensions and Selection of Engagement Emotion Regulation Strategies at the Within-Individual Level (texts shorter than 60 words were removed)*

| Disengagement Strategies | Experiential Avoidance | | | | | Expressive Suppression | | | | | Distraction | | | | |
| --- | --- | --- | --- | --- | --- | --- | --- | --- | --- | --- | --- | --- | --- | --- | --- |
|  | **Hyp** | OR | SE | 95% CI | *p* | **Hyp** | OR | SE | 95% CI | *p* | **Hyp** | OR | SE | 95% CI | *p* |
| 1. Negative Valence | **+ (>1)** | 1.42 | 0.35 | [0.88, 2.30] | .16 | **+ (>1)** | 0.75 | 0.14 | [0.52, 1.09] | .13 | **+ (>1)** | 1.25 | 0.16 | [0.96, 1.62] | .09 |
| 2. Self-Focus | **+ (>1)** | 1.45 | 0.31 | [0.95, 2.19] | .08 | **+ (>1)** | 0.99 | 0.19 | [0.67, 1.45] | .95 | **+ (>1)** | 0.99 | 0.12 | [0.77, 1.26] | .93 |
| 3. Discrepancies | **+ (>1)** | 0.96 | 0.18 | [0.67, 1.38] | .81 | **+ (>1)** | 1.09 | 0.20 | [0.75, 1.57] | .65 | **+ (>1)** | 0.96 | 0.11 | [0.77, 1.21] | .74 |
| 4. Certitude | **E** | 0.89 | 0.16 | [0.62, 1.27] | .51 | **E** | 0.98 | 0.16 | [0.71, 1.36] | .91 | **E** | **1.38** | **0.17** | **[1.08, 1.76]** | **.01** |
| 5. Interpersonal | **E** | 0.97 | 0.19 | [0.65, 1.43] | .86 | **E** | 0.79 | 0.14 | [0.56, 1.11] | .17 | **E** | 0.85 | 0.10 | [0.67, 1.08] | .19 |
| 6. Past Focus | **+ (>1)** | 0.96 | 0.17 | [0.68, 1.37] | .82 | **+ (>1)** | 0.85 | 0.13 | [0.63, 1.16] | .31 | **+ (>1)** | 1.17 | 0.14 | [0.93, 1.48] | .19 |
| 7. Future Focus | **E** | 1.30 | 0.27 | [0.86, 1.96] | .21 | **E** | 1.39 | 0.28 | [0.93, 2.07] | .11 | **E** | 0.89 | 0.11 | [0.71, 1.12] | .33 |
| 8. Repetitiveness | **+ (>1)** | 1.22 | 0.26 | [0.81, 1.86] | .34 | **+ (>1)** | 1.34 | 0.23 | [0.95, 1.88] | .10 | **+ (>1)** | **1.29** | **0.16** | **[1.01, 1.63]** | **.04** |
| Engagement Strategies | Reappraisal | | | | | Problem solving | | | | | Acceptance | | | | |
|  | **Hyp** | OR | SE | 95% CI | *p* | **Hyp** | OR | SE | 95% CI | *p* | **Hyp** | OR | SE | 95% CI | *p* |
| 1. Negative Valence | **– (<1)** | 0.75 | 0.14 | [0.52, 1.09] | .13 | **– (<1)** | 1.09 | 0.11 | [0.89, 1.34] | .42 | **– (<1)** | 0.88 | 0.09 | [0.72, 1.07] | .21 |
| 2. Self-Focus | **– (<1)** | 0.99 | 0.19 | [0.67, 1.45] | .95 | **– (<1)** | 1.05 | 0.11 | [0.85, 1.28] | .66 | **– (<1)** | 0.92 | 0.09 | [0.75, 1.12] | .42 |
| 3. Discrepancies | **– (<1)** | 1.09 | 0.20 | [0.75, 1.57] | .65 | **– (<1)** | 0.92 | 0.09 | [0.76, 1.11] | .37 | **– (<1)** | 0.99 | 0.09 | [0.82, 1.19] | .89 |
| 4. Certitude | **+ (>1)** | 0.98 | 0.16 | [0.71, 1.36] | .91 | **– (<1)** | 0.86 | 0.08 | [0.72, 1.04] | .12 | **+ (>1)** | 0.96 | 0.09 | [0.81, 1.15] | .69 |
| 5. Interpersonal | **E** | 0.79 | 0.14 | [0.56, 1.11] | .17 | **E** | **0.58** | **0.06** | **[0.48, 0.71]** | **<.001** | **E** | 1.05 | 0.10 | [0.87, 1.28] | .59 |
| 6. Past Focus | **+ (>1)** | 0.85 | 0.13 | [0.63, 1.16] | .31 | **– (<1)** | **0.81** | **0.08** | **[0.68, 0.98]** | **.03** | **+ (>1)** | **1.22** | **0.12** | **[1.00, 1.48]** | **.045** |
| 7. Future Focus | **– (<1)** | 1.39 | 0.28 | [0.93, 2.07] | .11 | **+ (>1)** | 0.99 | 0.10 | [0.80, 1.22] | .93 | **– (<1)** | 1.08 | 0.10 | [0.89, 1.30] | .45 |
| 8. Repetitiveness | **– (<1)** | 1.34 | 0.23 | [0.95, 1.88] | .10 | **– (<1)** | 0.98 | 0.10 | [0.80, 1.20] | .86 | **– (<1)** | 1.13 | 0.11 | [0.94, 1.37] | .20 |

*Note*. “+ (>1)” denotes significant positive associations where OR is larger than 1 in hypotheses, “– (<1)” denotes significant negative associations where OR is less than 1 in hypotheses, “E” denotes Exploratory in hypotheses.

Hyp = Hypothesis OR = Odds Ratio. SE = Standard Error of Odds Ratio. 95% CI = 95% Confidence Interval of Odds Ratio; Coefficients were calculated based on standardized data.
